# Could Local Dynamic Stability Serve as an Early Predictor of Falls in Patients with Moderate Neurological Gait Disorders? A Reliability and Comparison Study in Healthy Individuals and in Patients with Paresis of the Lower Extremities

**DOI:** 10.1371/journal.pone.0100550

**Published:** 2014-06-20

**Authors:** Fabienne Reynard, Philippe Vuadens, Olivier Deriaz, Philippe Terrier

**Affiliations:** 1 Clinique romande de réadaptation SUVACare, Sion, Switzerland; 2 Institute for Research in Rehabilitation, Sion, Switzerland; Charité University Medicine Berlin, Germany

## Abstract

Falls while walking are frequent in patients with muscular dysfunction resulting from neurological disorders. Falls induce injuries that may lead to deconditioning and disabilities, which further increase the risk of falling. Therefore, an early gait stability index would be useful to evaluate patients in order to prevent the occurrence of future falls. Derived from chaos theory, local dynamic stability (LDS), defined by the maximal Lyapunov exponent, assesses the sensitivity of a dynamic system to small perturbations. LDS has already been used for fall risk prediction in elderly people. The aim of the present study was to provide information to facilitate future researches regarding gait stability in patients with neurological gait disorders. The main objectives were 1) to evaluate the intra-session repeatability of LDS in patients and 2) to assess the discriminative power of LDS to differentiate between healthy individuals and neurological patients. Eighty-three patients with mild to moderate neurological disorders associated with paresis of the lower extremities and 40 healthy controls participated in the study. The participants performed 2×30 s walking wearing a 3D accelerometer attached to the lower back, from which 2×35 steps were extracted. LDS was defined as the average exponential rate of divergence among trajectories in a reconstructed state-space that reflected the gait dynamics. LDS assessed along the medio-lateral axis offered the highest repeatability and discriminative power. Intra-session repeatability (intraclass correlation coefficient between the two repetitions) in the patients was 0.89 and the smallest detectable difference was 16%. LDS was substantially lower in the patients than in the controls (33% relative difference, standardized effect size 2.3). LDS measured in short over-ground walking tests seems sufficiently reliable. LDS exhibits good discriminative power to differentiate fall-prone individuals and opens up the possibility of future clinical applications for better prediction of fall risk in neurological patients.

## Introduction

In patients with muscular dysfunction associated with a central nervous system pathology, the maintenance of optimal mobility in daily living activities is of utmost importance [1], [2]. Unfortunately, those patients frequently exhibit altered walking capabilities, even in the case of mild disorders [3], [4], [5]. The basic requirements for walking, which are the capabilities to maintain a stable upright posture and to initiate and maintain rhythmic stepping, are compromised. Gait pattern, even if functional, remains different from a normal pattern, and kinematic, kinetic and electromyographic characteristics of the trunk and the lower extremities can be disturbed [6], [7], [8], [9]. Thus, those gait disorders are associated with a higher risk of falling [10], [11]. For instance, about 40% of people fall within the first year after a stroke [12]. A high yearly incidence (>50%) of falls among people with multiple sclerosis or traumatic brain injury has also been reported [13], [14], [15]. This incidence increases to as high as 75% in individuals with incomplete spinal cord injury [16].

The occurrence of a single fall can trap the patient in a vicious circle: the injuries due to the fall can lead to transient disabilities, deconditioning, and fear of falling, which decrease walking capabilities and hence further increase the risk of falling. Consequently, it is of paramount importance to identify patients at high risk of falling even before falls are reported. Furthermore, practical and efficient early detection must rely on rapid and simple tests that can be administered by medical staff in clinical settings. An interesting candidate to fulfill those requirements is local dynamic stability (LDS). This index, derived from chaos theory, assesses the sensitivity of gait to small perturbations that occur naturally during walking, due to either internal causes (neuromuscular noise), or small external disturbances (such as floor irregularities). LDS can be estimated from a simple measure of gait dynamics, such as trunk acceleration [17]. Recent theoretical [18], [19] and experimental [20], [21] results have shown that LDS can serve as an early indicator for falling risk [22]. In addition, it is very likely that LDS can differentiate among fallers and non-fallers in elderly people [11], [23]. We [24] and others [25] have very recently provided evidence that LDS can be reliably assessed with short walking tests that are suited for patients with low walking capabilities. Two of our recent studies showed that LDS could be used in clinical settings [26], [27]. We also found that LDS assessed using the medio-lateral trunk acceleration and over the average duration corresponding to one step offered the highest reliability in treadmill walking [24], but these findings still need confirmation in over-ground walking experiments. Finally, there is a lack of information regarding the applicability of LDS in patients with neurological disorders, especially in young and middle-aged individuals.

The objective of the present study was to provide preliminary information to facilitate future research on gait stability –assessed with LDS– in patients with neurological gait disorders. We studied 40 healthy individuals and 83 patients with mild to moderate neurological gait disorders associated with paresis of the lower extremities. Tridimensional trunk acceleration was measured during short over-ground walking tests. First, in order to complement the results obtained in treadmill walking [24], we evaluated the intra-session repeatability of LDS in both healthy controls and patients (reliability study). Second, we aimed to provide reference values for LDS in patients and to assess the degree to which those values differ from those of healthy controls (comparison study). Third, we sought to assess which methodology (LDS estimated over one stride or over one step) and which axis (medio-lateral, vertical, or antero-posterior) would be the best suited for assessing neurological patients. Finally, we analyzed the relationship between LDS and basic temporal gait parameter (cadence) with the purpose of determining how much LDS variance could be explained by the gait pattern variability among individuals.

## Methods

### Participants

Patients who underwent a gait analysis between July 2007 and November 2013 were retrospectively selected from the database of the clinical gait analysis laboratory at the Clinique romande de réadaptation (Sion, Switzerland). The patients consulted because of gait disorders related mainly to paresis of various etiologies. In addition to a short walking test, the patients underwent several analyses (mobility of the lower limbs, electomyography), carried out by the same team of physiotherapist and supervised by two skilled neurologists. Inclusion criteria were (1) less than 65 years of age; (2) diagnosed with a central nervous system disorder; (3) reduced walking capabilities; and (4) able to walk independently and continuously for one minute at a minimal cadence of 84 steps/minute, which corresponds to 35 steps in 25 seconds. Among 260 eligible patients, 83 met the inclusion criteria. The reasons for exclusion were (in order of importance): (1) incomplete data (mostly due to organizational issues); (2) insufficient walking capability (low speed, frequent stops); (3) age; (4) inappropriate pathology (e.g., peripheral disorders as primary diagnosis). The included patients, whose characteristics are summarized in Table 1, suffered from stroke (N = 24), multiple sclerosis (N = 12), traumatic brain injury (N = 10), cerebral palsy (N = 15), spinal cord injury (N = 18), or hereditary spastic paraplegia (N = 4). They exhibited muscle spasticity of the lower extremities (N = 12), strength loss of the lower extremities (N = 21), or combined strength loss and spasticity (N = 34). The remaining patients (N = 16) had near normal force and no relevant spasticity, however they presented mild gait dysfunctions related to their pathologies.

**Table 1:** Participants' characteristics.

	Patients N = 83	Healthy controls N = 40
men/women	48/35	20/20
age (yr.)	44 (14)	40 (9)
height (m)	1.70 (0.09)	1.72 (0.08)
weight (kg)	71 (15)	69 (13)
time since lesion (yr.)	14 (14)	-

The values are means (SD).

In order to compare the patients with healthy controls, we analyzed 40 healthy individuals; their characteristics are also reported in Table 1. The walking tests of most of the controls (36/40) had already been analyzed in another study [28]. All subjects gave their written informed consent for the use of their data for research purposes. The study was approved by the regional medical ethics committee (Commission d'éthique du Valais, Sion, Switzerland).

### Walking test

During a short walking test, trunk accelerations were recorded with a tri-axial accelerometer that was attached with an elastic belt at the level of the L3–L4 spinous process and connected to a lightweight data logger (Physilog system, GaitUp, Lausanne, Switzerland; sampling rate 200 Hz, 16-bit resolution). The accelerometer measured the body accelerations along three axes: medio-lateral (ML), vertical (V) and antero-posterior (AP). The patients were instructed to walk straight ahead, while barefoot, at a self-selected comfortable walking speed, along a 70-meter hallway for a period of at least 30 seconds. The measurement was performed twice, wearing the accelerometer continuously in order to ensure a consistent placement of the sensor. The same procedure was carried out with the healthy controls. The subsequent data analysis was performed with Matlab R2013a (MathWorks, Natick, MA).

### Data pre-processing

The first 5 s of the raw acceleration data were discarded to rule out starting effects. In order to mitigate sensor placement variability among participants, the 3D-acceleration signals were reoriented according to a standard procedure [24], [29]. From the following 25 s, the cadence (or step frequency, SF), expressed in steps per second (Hz), was computed with a fast Fourier transform algorithm using the vertical acceleration signal. Then, a uniform number of steps (35) were kept for the subsequent analysis. The 35-step signals were then time-normalized to a uniform length of 4000 samples using a polyphase filter implementation.

### Local dynamic stability

We thoroughly described the theoretical background behind LDS methodology in a recent study [30]. In short, the method examines structural characteristics of a time series embedded in an appropriately reconstructed state-space that reflects the dynamics of the system. The reconstruction relies on the fact that the characteristics of a dynamic system, defined by several interdependent differential equations, can be depicted by analyzing a single variable and several time-delayed copies of itself (Takens' theorem [30], [31]). In practice, the time delay was assessed with an average mutual information (AMI) analysis [30], [32], and the number of embedding dimensions with a global false nearest neighbours (GFNN) analysis [30], [33]. According to the average results of those analyses, a uniform delay (number of samples: ML = 12, V = 16, AP = 19), and a constant dimension (6) were used [34]. In order to estimate the sensitivity to infinitesimal perturbations (“butterfly effect” in chaos theory), the increasing distance between trajectories (i.e., divergence) downstream from initially nearby points in the state-space was computed, as proposed by Rosenstein et al. for small data sets [35]. This method estimates the maximum finite-time Lyapunov exponent (λ) from the slope of linear fit in the logarithmic divergence diagram (time vs. divergence) [17], [30]. Although human walking is probably not a pure, low-dimensional, chaotic system that can be characterized by Lyapunov exponents, the slopes (exponents) can quantify the average rate of exponential divergence (and hence, local stability) among state-space trajectories (representative of gait dynamics) at different time scales. In the present study, divergence exponents along each axis (ML, V, and AP) were computed either over the duration of one step downstream from nearest neighbors in the state-space (λ_0.5_) or over the duration of one stride (λ_1_). Because of the resampling to a uniform sample length, a constant number of samples corresponding to one step (114) or one stride (228) was used for the linear fitting.

### Statistics

Seven dependent variables were analyzed in the patients (N = 83) and the healthy controls (N = 40): cadence (or step frequency); LDS computed over one step (divergence exponent λ_0.5_) in ML, V and AP directions; and LDS computed over one stride (divergence exponent λ_1_) in ML, V and AP directions.

**Figure 1:**
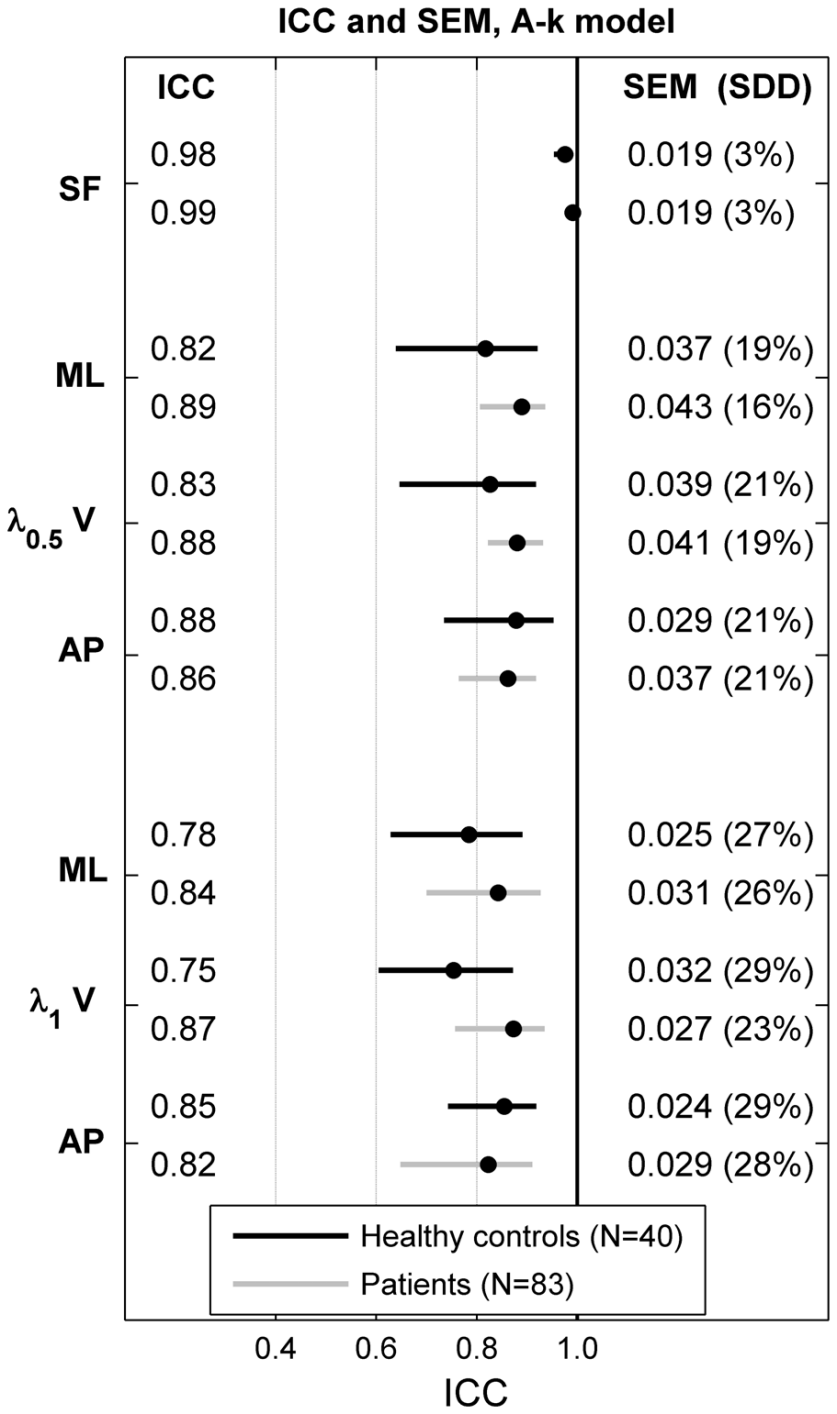
Intrasession reliability of local dynamic stability. Forty healthy individuals (Healthy controls) and 83 patients exhibiting mild to moderate neurological disorders (Patients) walked 2×30 sec. at preferred speed. A 3D accelerometer recorded the trunk acceleration in medio-lateral (ML), vertical (V) and antero-posterior (AP) directions. The cadence (or step frequency, SF) was assessed by spectral analysis of the vertical acceleration signal. Local dynamic stability was evaluated by computing the rate of the average divergence among nearby trajectories in a reconstructed state-space that reflects the dynamics of locomotion (Lyapunov exponent method). The average divergence was computed either over one step (λ_0.5_) or over one stride (λ_1_). The absolute agreement among the two repetitions by intraclass correlation coefficient (ICC(A,k), the standard error of measurement (SEM), and the relative smallest detectable difference (SDD) are shown. The 95% confidence intervals (CI) were computed by bootstrapping (5000 resamples).

The relative (intraclass correlation coefficient, ICC) and absolute (standard error of measurement, SEM) intra-session repeatability were assessed for each dependent variable, separately for healthy controls and patients (Figure 1). The ICC method was that proposed by McGraw and Wong [36]. The two 35-step walking tests were used as two within-subject repetitions. The ICC (A,k) model was used, which assesses the degree of agreement among measurements, when the average over measurements is considered. In addition, we computed 95% confidence interval on ICC values using bootstrapping (5000 resamples, bias corrected and accelerated percentile method). The SEM is the group-level estimation of the within-subject average variability [37], defined by the following equation: 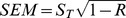, where *S* 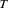 is the global standard deviation and *R* is the corresponding ICC. From SEM, we also computed the smallest detectable difference (*SDD* = *SEM×1.96*×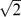), which is the smallest change that could be considered significant [37]. Normalized by the mean and expressed as percentage, it allows evaluation of whether an observed relative change in one individual is not due to measurement error or to intra-individual variability.

**Figure 2:**
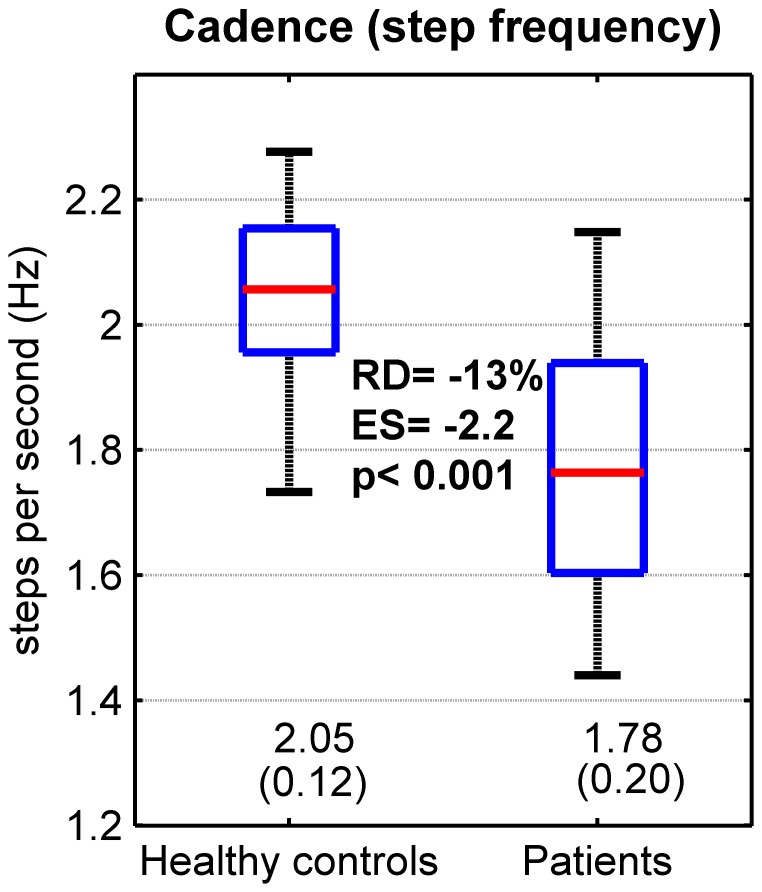
Cadence: descriptive statistics and comparisons between healthy controls and patients. Forty healthy individuals (Healthy controls) and 83 patients exhibiting mild to moderate neurological disorders (Patients) walked 2×30 sec. at preferred speed. A 3D accelerometer recorded the trunk acceleration. The cadence (or step frequency) was assessed by spectral analysis (Fourier transform) of the vertical acceleration signal. The results of the two 30sec. walking bouts were averaged. The spread of the data among participants is presented with boxplots (median and quartiles). Mean and SD are shown at the bottom of the figure. The bold values are as follows: top: the relative difference (RD) between controls and patients, expressed as percentage; middle: effect size (ES), i.e., mean difference normalized by SD of the controls (Glass's delta); p: p-value of the t-test between controls and patients (unequal variance).

**Figure 3:**
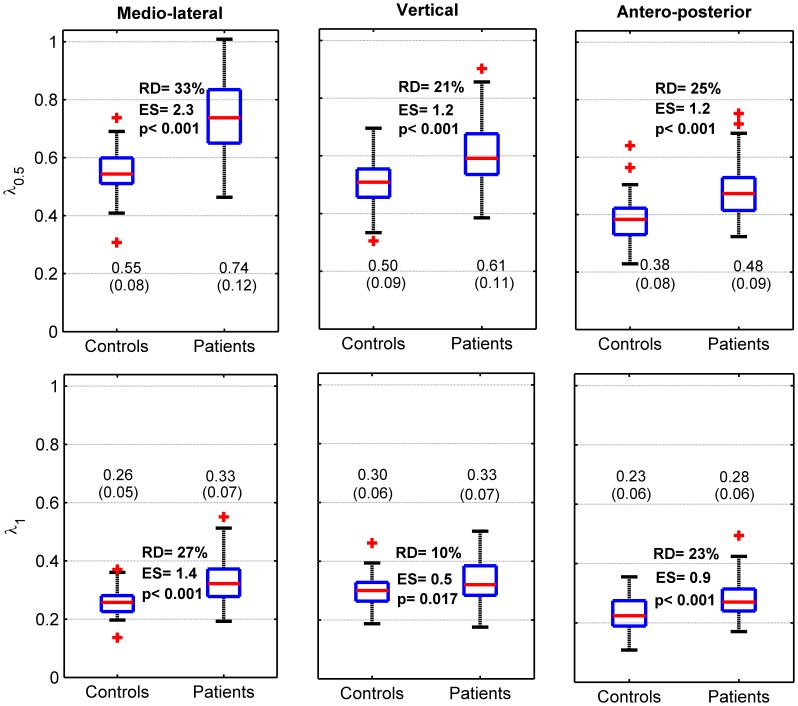
Local dynamic stability: descriptive statistics and comparisons between healthy controls and patients. Forty healthy individuals (Controls) and 83 patients exhibiting mild to moderate neurological disorders (Patients) walked 2×30 sec. at preferred speed. A 3D accelerometer recorded the trunk acceleration in medio-lateral (ML), vertical (V) and antero-posterior (AP) directions. Local dynamic stability was evaluated by computing the rate of the average divergence among nearby trajectories in a reconstructed state-space that reflects the dynamics of locomotion (Lyapunov exponent method). The divergence was computed either over one step (λ_0.5_) or over one stride (λ_1_). The results of the two 30sec. walking bouts were averaged. The spread of the data among participants is presented with boxplots (median, quartiles and outliers (red +)). Mean and SD are shown at the bottom of the individual panels. The bold values are as follows: top: the relative difference (RD) between controls and patients, expressed as percentage; middle: effect size (ES), i.e. mean difference normalized by SD of the controls (Glass's delta); p: p-value of the t-test between controls and patients (unequal variance).

**Figure 4:**
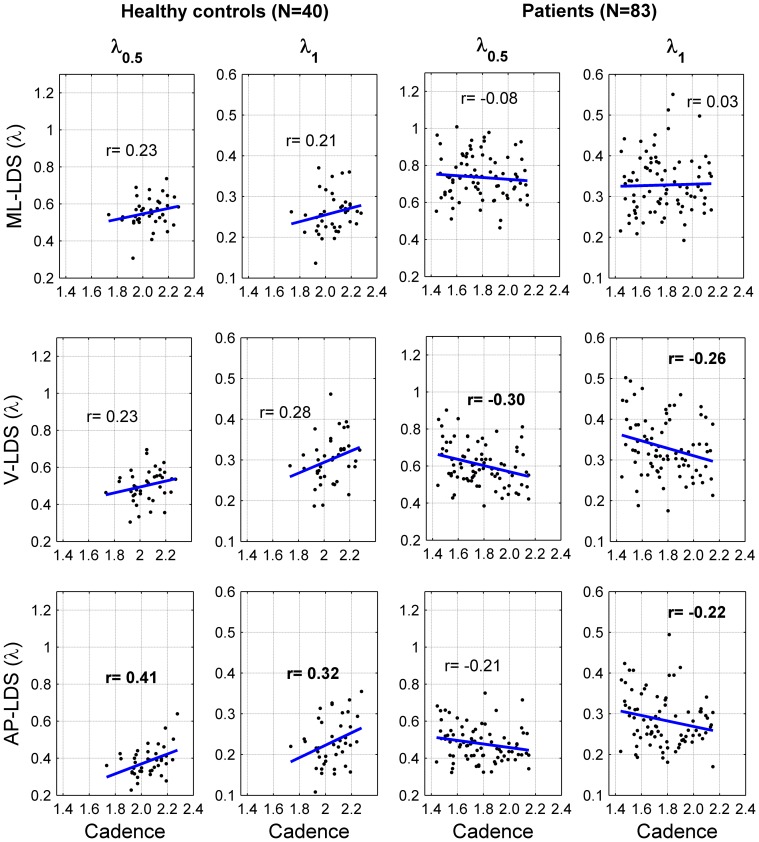
Scatter plots and correlations between cadence and local dynamic stability. Forty healthy individuals (Controls) and 83 patients exhibiting mild to moderate neurological disorders (Patients) walked 2×30 sec. at preferred speed. A 3D accelerometer recorded the trunk acceleration in medio-lateral (ML), vertical (V) and antero-posterior (AP) directions. The cadence was assessed by spectral analysis of the vertical acceleration signal. Local dynamic stability (LDS) was evaluated by computing the rate of the average divergence among nearby trajectories in a reconstructed state-space that reflects the dynamics of locomotion (Lyapunov exponent method). The divergence was computed either over one step (λ_0.5_) or over one stride (λ_1_). The results of the two 30sec. walking bouts were averaged. The printed values are Pearson's correlation coefficient. Bold values indicate significant correlation (r≠0). Blue lines are the linear best fits (least squares method).

The two within-subject repetitions were then averaged for the other analyses. Boxplots (median and quartiles), means and standard deviations (SD) were used to describe the distribution of the dependent variables among participants (Figure 2 and 3). Two parameters were computed to estimate the differences between the healthy controls (N = 40) and the neurological patients (N = 83): 1) relative difference, (patients – controls)/controls *100 and 2) standardized effect size (or standardized mean difference), using the Glass's delta (i.e. average difference (patients – controls) divided by the SD of the controls [38]). Statistical significance was inferred using unpaired t-tests, assuming unequal variance between groups. In order to take into account the inflated type I error risk due to multiple comparisons (six t-tests), the Bonferroni correction was applied: the significance level corresponding to 5% was p = 0.008. Finally, we analyzed the strength of the relationship between cadence (SF) and LDS, using Pearson's r correlation coefficient. The r coefficients were computed separately for the healthy controls and the patients (Figure 4).

## Results

The reliability results (Figure 1) show that LDS measured over one step (λ_0.5_) exhibits a higher repeatability than LDS measured over one stride (λ_1_), both in terms of relative reliability (average ICC, 0.86 vs. 0.82) and in terms of absolute reliability (SEM and average SDD, 20% vs. 27%). The neurological patients presented higher ICCs than the healthy controls, mainly due to the larger inter-individual variability among them (see Figure 2 and 3). The smaller confidence intervals in the patients are due to the larger sample size. On the other hand, the SDDs were mostly comparable between the controls and the patients. The most reliable stability parameter seemed to be λ_0.5_ measured in the medio-lateral direction, with high ICC in the patients (0.89), and particularly low SDD in the controls and patients (19% and 16%, respectively).

As showed in Figure 2, the patients walked at a slower cadence than the healthy individuals did (relative difference, -13%). In addition, there was more inter-individual variability among the patients (coefficient of variation: 11%) than among the controls (6%).

Figure 3 presents descriptive statistics for LDS and shows the differences between the patients and the healthy individuals. It is important to remember that higher divergence exponents (λ) indicate lower stability. A higher contrast was given by the medio-lateral estimate of λ_0.5_, which indicates that the dynamic stability was reduced by 33% in the neurological patients. On the other hand, λ_1_ exhibited a smaller difference, with, for example, only 10% of relative difference in the vertical direction.

The correlation analysis (Figure 4) highlights that the cadence and the LDS were rather loosely related: the average r^2^ is 6%. Furthermore, over 12 correlations only five were significant. Nonetheless, a trend to a positive association between cadence and LDS is apparent in healthy controls. It is also possible that in the patients, there was a negative association between the LDS measured along the vertical axis and the cadence.

## Discussion

This study analyzed gait stability in 40 healthy controls and 83 neurological patients, who performed a short walking test (about one minute) wearing a 3D accelerometer that measured trunk acceleration. Gait stability was assessed using a methodology derived from chaos theory (divergence exponent that reflects local dynamic stability). In summary, the results indicate a good repeatability of the LDS (ICC mostly between 0.8 and 0.9, Figure 1). The discriminative power of LDS to differentiate between controls and patients was high (effect size 0.5–2.3; Figure 3). LDS measured along the medio-lateral direction and over one step (λ_0.5_-ML) is likely the most suitable stability index, given the reliability (Figure 1) and the effect size (Figure 3). Furthermore, the strength of the association between LDS and cadence was rather low among individuals (Figure 4), which likely indicates that cadence variability in population should not constitute a relevant confounding factor to identify individuals at risk of falling. Overall, the results provide conclusive evidence that the LDS methodology is suitable for evaluating the gait stability of neurological patients in a clinical setting. Nevertheless, the association between gait stability and actual fall rate remains to be investigated.

The inclusion criteria of the present study were chosen in order to select patients with a good walking capacity, as this population is precisely the target for early evaluation of falling risk. Although the included patients suffered from different pathologies, the common denominator among them was that they exhibited paresis and/or spasticity of the lower limbs, related mainly to disorders of the corticospinal (pyramidal) tract. This clinical picture is found in a substantial part of the neurologically impaired population with gait disorders; however, other pathologies (e.g., extrapyramidal disorders, such as Parkinson's disease or ataxia) should be investigated in future studies.

Several studies have highlighted decreased gait stability in older age [39], [40], associated with increased fall risk [11], [23], which explains why older patients (age>65 yr.) were excluded from the present analysis. Although age was not perfectly identical between the healthy controls and the patients, the difference was sufficiently small (four years) to have no substantial effect.

A methodological purpose of the present study was to provide new information to help choose between two methods used to compute divergence exponents. Actually, two time scales have been proposed to perform the linear fit in the divergence diagrams, either over one step downstream of the nearest neighbors in the state-space (λ_0.5_ [11], [34]), or over one stride (λ_1_ [41], [42]). In a previous study of 95 healthy individuals performing treadmill walking [24], we observed that λ_0.5_ exhibited better precision. The use of λ_0.5_ is further supported by the evidence presented here: it is confirmed (Figure 1) that for over-ground walking, in both healthy individuals and neurological patients, λ_0.5_ offers better reliability. Moreover, λ_0.5_ is more sensitive in differentiating between patients and controls (Figure 3).

The use of wearable motion sensors in the field of rehabilitation has attracted growing interest [43], and accelerometers are used routinely for physical activity monitoring [44]. Due to their ease of use and relatively low cost, those instruments are ideal for performing rapid gait evaluation in a clinical context [26], [27], especially because it has been shown that they constitute a valid alternative in assessing LDS compared to an optoelectronic measurement system [45]. We acknowledge that collecting supplementary gait parameters (speed, step width, temporal pattern etc.) can provide additional information to document the gait disorder and potentially detect fall risk, but at the cost of more complicated measurement process. Although we used a high accuracy accelerometer (200 Hz/16 bit sampling) in the present study, the capabilities of simpler and cheaper accelerometers, such for instance the new Actigraph GT3X+ (ActiGraph, Pensacola, FL) [46], might be sufficient (100 Hz/12bit sampling) for recording trunk acceleration for stability analysis. In this context, we seek to establish whether the use of a single-axis measure of trunk acceleration could be suitable to simplify the instrumentation (single-axis accelerometers would be sufficient) and to facilitate the interpretation of the results; there is strong evidence that the medio-lateral axis is the best candidate for that purpose. Indeed, it offers the best reliability for both healthy controls (relative SDD 19% vs. 21%, Figure 1) and patients (16% vs. 19%–21%). We drew the same conclusion in our previous study in healthy individuals performing treadmill walking [24]. In addition, LDS in the medio-lateral axis seems more sensitive for differentiating between healthy individuals and patients than the other axes (relative difference 33% vs. 21%–25%; Figure 3). Finally, theoretical considerations support the use of frontal-plane dynamics to characterize gait stability. An active motor control is very likely necessary in this plane to stabilize gait, while other directions would be constitutively stable [47]. As a result, impaired motor control, which could lead to falls, is potentially easier to detect by measuring acceleration along that direction.

Given these methodological considerations, λ_0.5_ measured in the medio-lateral direction is the parameter that we advise using for further gait stability studies in clinical settings. The results (Figure 3) can be used as a reference scale against which to compare patients: the 95% confidence intervals are 0.40–0.71 in healthy individuals and 0.50–0.98 in neurological patients. However, it must be stated that the absolute value of LDS is influenced by the methodology (among others, sample length [24]) and thus, those references are applicable to 35-step tests (or the average of several 35-step tests) in which the trunk acceleration is measured at the lower back level.

In 2006, in the first study to analyze the intra-session reliability of gait LDS, Kang and Dingwell [48] showed that 2–3 minute treadmill tests were necessary to obtain good precision (ICC>0.75). In 2009, by analyzing a small sample of young individuals (N = 9), Bruijn and colleagues [49] reported that at least 150 consecutive strides were likely required to obtain sufficient sensitivity and precision. The results of Van Schooten and colleagues [50], published in 2011, suggested that reliable LDS estimates could be obtained by averaging the results of several small walking bouts (10 m). By analyzing long duration outdoor walking (200 strides) in young healthy subjects (N = 20), the same research group reported [34] good intrasession repeatability (ICC: 0.79–0.92) and lower intersession (two non-consecutive days) repeatability (ICC: 0.38–0.63). In the abovementioned study, which analyzed 95 healthy individuals during treadmill walking [24], we observed that the ICC and SDD for 35-stride length estimates of λ_0.5_ measured in the medio-lateral direction were 0.74 and 15%. As a comparison, in the present study, under over-ground walking conditions, we reported values of 0.82 and 19% (figure 1). Although the ICC values were higher in the neurological patients than in the controls (0.89 vs. 0.82) due to the larger between-subjects variability (Figure 3), the SDD values were comparable (16% vs. 19%); therefore, neurological conditions likely do not increase within-subject LDS variability. This SDD result suggests that it would be difficult to evaluate small LDS changes at the individual level. However, it has been obtained by analyzing only one minute walking. By averaging eight 35-steps tests (about four minute of walking), the predicted SDD would be 8% [24], [28]. On the other hand, the results only reflect intra-session repeatability. Because lower inter-session (between days) repeatability has been reported [24], [34], several LDS estimates measured on different days should probably be relied on in longitudinal studies [24].

Regarding the comparison study, a high contrast (33%, ES = 2.3, Figure 3) was observed between the healthy individuals and the neurological patients. To put this finding in perspective, the following results were reported in the literature. In a small sample of subjects (N = 8), Lockhart and Liu [23] found that fall-prone elderly individuals exhibited higher divergence exponents (i.e., lower stability) than healthy counterparts; the relative difference was between 20% and 31%. Buzzi and colleagues [39] observed that the dynamic stability of young adults (N = 10, mean age 25 years) differed substantially (22%–44%) from that of elderly individuals (N = 10, mean age 75 years). Similarly, Kang and Dingwell [40] reported about 50% relative difference in LDS between healthy older adults (N = 18, age 65–85 years) and younger matched controls (N = 18, age 18–20 years). Recently, Huisinga and colleagues [51] found a substantially lower LDS in patients with multiple sclerosis (N = 15) as compared to matched controls (N = 15): the relative difference was about 40%. Obtained in a large sample (40 controls, 83 patients), the results of the present study are in line with previous studies: because LDS strongly contrasts between healthy controls and fall-prone individuals, it likely constitutes a sensitive indicator of gait instability and fall risk. However, further longitudinal studies are needed to confirm whether the actual fall rate is related to LDS in neurological patients, as already suggested for elderly individuals [11], [23].

It has been shown that gait LDS could be modified by constraining gait kinematics. The simplest constraint is to impose different speeds on a treadmill, which has substantial effect on LDS [40], [52], [53], [54]. Constraining step length [41] and cadence [55] also affects LDS. Therefore, at first sight, it is not excluded that difference between groups, such as depicted in Figure 3, is the result of a difference in gait kinematics and not a difference in gait stability, even more considering that a difference in cadence exists (Figure 2). We performed the correlation analysis (Figure 4) to exclude that possibility. We used cadence as a simple kinematic parameter, because it can be computed directly from the same acceleration signal as LDS. Furthermore, both cadence and step length increase/decrease in parallel to produce speed modifications [56]. As a result, cadence is a proxy for walking speed. If we focus on the proposed reference value (λ_0.5_ – ML), there is no correlation between cadence and LDS in patients (r = −0.08). In other words, if a patient exhibits a high cadence (and hence, likely a high walking speed), nothing can be predicted about his/her dynamic stability. Therefore, LDS is effective for measurement and cadence does not constitute a confounding factor. This finding should be put into perspective with the results of our recent study, which showed that LDS was more responsive (larger effect size) than cadence or speed in discriminating therapeutic effects in patients with multiple sclerosis [27]. On the other hand, a small positive correlation exists in healthy individuals (r = 0.23). Individuals, who walk spontaneously with a slower cadence (and likely a slower preferred walking speed), tend to be *more* stable than individuals, who walk with a faster cadence. Similar speed dependence has been described in treadmill experiments [40], [52]. Conversely, neurological patients, who walked with s slower cadence (−13%, Figure 2), were *less* stable (higher λ, +33%, Figure 3). This finding likely excludes that kinematic difference between healthy individuals and patients explain LDS differences.

## Conclusions

For routine analysis of gait stability in patients by medical staff, (1) the measure should be simple to perform and the computing should be straightforward, and (2) the results should be reliable (low intra-individual variability), (3) valid (representative of the investigated issue) and (4) easy to interpret (single value with clear meaning). Given those requirements, the present study provides the following information.

1. Short walking tests are easy to perform, and are short enough (about one minute) not to exacerbate pain and to keep a low level of fatigue; furthermore, the accelerometer is easy to attach (simple belt) and to use (start/stop button). However, the computing of LDS still requires custom software and programing skills.
2. The reliability of LDS, estimated in healthy controls as well as in neurological patients, seems sufficient to assess small differences between groups. However, longer walking tests (more repetitions) are needed to detect small changes at the individual level. Furthermore, given the substantial day-to-day variability of LDS [24], [34], it should be measured on different days to further improve reliability.
3. Although no true validation analysis was performed (e.g., strength of the association between LDS and actual fall rate), the results show that LDS can differentiate between healthy individuals and neurological patients with a high confidence (effect size 2.3). Furthermore, as highlighted by the correlation results (cadence vs. LDS), kinematic variability among individuals does not seem to constitute a relevant confounding factor.
4. It is likely that short-term LDS measured over one step (λ_0.5_) in the medio-lateral direction is the best candidate to provide a single value to evaluate gait stability. Provided that the same methodology is used (LDS measured over 35 steps, accelerometer attached to the lower back), the results (Figure 3) could be used as a reference stability scale, to which individual LDS results could be compared.

It has been shown that specific management programs and exercise interventions can reduce fall risk in older individuals [57]. We showed that a three week rehabilitation program can improve static and dynamic stability in patients with multiple sclerosis [27]. Consequently, early identification of patients at risk for falling is needed in order to improve therapeutic management. Although further studies are needed to confirm its validity, LDS seems an appropriate tool to achieve this goal.

### Data accessibility

Due to ethical policy, the access to raw individual data has been restricted. However, the data can be made available upon request through academic cooperation.
